# Exploring the impact of AI on unemployment for people with disabilities: do educational attainment and governance matter?

**DOI:** 10.3389/fpubh.2025.1559101

**Published:** 2025-04-02

**Authors:** Anis Omri, Henda Omri, Hatem Afi

**Affiliations:** ^1^Department of Business Administration, College of Business and Economics, Qassim University, Qassim, Saudi Arabia; ^2^King Salman Center for Disability Research, Riyadh, Saudi Arabia; ^3^Higher Institute of Business Administration, University of Gafsa, Gafsa, Tunisia; ^4^Department of Accounting, College of Business and Economics, Qassim University, Qassim, Saudi Arabia

**Keywords:** unemployment, artificial intelligence, education, governance, moderated mediation model

## Abstract

The current study investigates the impact of artificial intelligence (AI) on unemployment among people with disabilities, focusing on the mediating role of education and the moderating effect of governance quality. Using panel data from 27 high-tech developed countries between 2006 and 2022, the findings reveal a nuanced relationship where AI initially increases unemployment among people with disabilities due to automation and skill mismatches. However, advanced education mitigates this effect, significantly improving employability by equipping individuals with market-relevant skills. Governance quality plays a critical role in this dynamic, amplifying AI’s positive impact on education while, paradoxically, intensifying its negative effects on unemployment when governance frameworks are weak or misaligned. These findings underscore the importance of robust governance structures and targeted educational initiatives to harness AI’s potential in fostering inclusive labor markets. Policymakers should align AI investments with governance reforms and education systems to ensure equitable employment opportunities for people with disabilities.

## 1 Introduction

Artificial intelligence has become increasingly recognized for its transformative impact on various aspects of society, including the labor market. By removing conventional barriers to work, artificial intelligence holds special promise for reducing unemployment among individuals with disabilities in high-tech developed nations. Due to things like restricted physical accessibility, rigid work conditions, and prejudices throughout the hiring process, it has historically been difficult for people with disabilities to find and keep a job (1). People with disabilities can participate fully in the workforce and take advantage of a broader range of opportunities thanks to AI-driven tools and applications that can lower these obstacles. Artificial intelligence not only makes it easier to find work, but it also promotes sustainable employment by enabling technologies such as intelligent job-matching systems, assistive AI-powered devices, and automated work platforms that allow tasks to be tailored to each worker’s particular needs and strengths. Moreover, the emergence of AI-based tools like voice recognition, screen readers, and sophisticated mobility aids improves workplace inclusivity by enabling people with cognitive, sensory, or physical disabilities to complete tasks that were previously challenging or unattainable (2). These developments lessen gaps in employment outcomes by empowering disabled people to actively engage in high-demand industries, such as those that demand technology capabilities.

Beyond immediate advancements in accessibility, artificial intelligence’s ability to enhance education and skill development is directly related to its potential to decrease unemployment rates for individuals with disabilities. In high-tech economies where the need for technology skills is critical, this is particularly relevant. Given the substantial correlation between educational attainment and employment rates and quality of employment, the relevance of education in preparing people with disabilities for the workforce cannot be overstated (3). Artificial intelligence can meet various learning demands via personalized and adaptive learning systems, providing specialized educational paths that can significantly enhance learning results for students with disabilities. AI-powered tools such as intelligent tutoring systems, for instance, can offer personalized learning experiences that adapt to each student’s learning style, strengths, and weaknesses (4). These systems use data to continuously evaluate a learner’s progress and provide feedback that helps pupils navigate complex material. Thanks to artificial intelligence in virtual learning settings, people with disabilities can now access education regardless of physical or geographical constraints. This is especially useful in sophisticated countries with well-established digital infrastructure. Because educational attainment lays the groundwork for essential job skills, which make individuals competitive in a tech-centric labor market, studies have demonstrated a direct correlation between artificial intelligence’s impact on education and increased employability among people with disabilities (5). By addressing educational inequalities and improving skills acquisition, artificial intelligence thus serves as a critical channel that links technology and employment for this demographic group.

However, artificial intelligence’s impact on employment and education is not an isolated phenomenon where governance plays a significant role. Good governance, characterized by effective laws, regulatory frameworks, and institutional support, is essential in high-tech developed countries to enable artificial intelligence’s beneficial effects on the education of persons with disabilities. The extent to which artificial intelligence applications are ethical, inclusive, and accessible is determined by governance, which establishes an environment where technological advances can successfully advance social and economic goals (6, 7). Good governance frameworks, for example, guarantee that AI-driven learning resources are used fairly, are free of algorithmic biases, and follow guidelines that safeguard the interests and rights of students with disabilities. According to Chan (8) and Moon (9), artificial intelligence in education is more likely to produce favorable results in areas where governance prioritizes inclusive policy frameworks. The accessibility and quality of educational materials for individuals with disabilities are improved by policies that support the financing and development of accessible technologies and programs that incorporate artificial intelligence into public education systems. Furthermore, a good governance climate encourages collaboration between government agencies, technology companies, and academic institutions, creating an ecosystem that adapts artificial intelligence applications to the needs of people with disabilities and ensures that educational and skills development opportunities are maximized for this group (10). Thus, governance serves as a moderator in the relationship between artificial intelligence and academic outcomes, highlighting that the degree to which the potential of artificial intelligence to improve employability through education can be realized depends on governance quality.

The conditional influence of governance quality on the indirect link between artificial intelligence and unemployment among those with disabilities is highlighted by this moderated mediation model. In particular, the model suggests that artificial intelligence’s impact on unemployment—mediated through educational attainment—is more effective in settings with robust governance structures. Studies, such as Crawford and Calo (11) and George and Wooden (12), show that the quality of governance affects educational institutions’ ability to successfully integrate AI-driven tools and the availability of these resources. Better educational outcomes that increase the employability of individuals with disabilities are made possible by good governance, which guarantees that artificial intelligence programs are inclusive and in line with ethical norms. On the other hand, the advantages of artificial intelligence in education might not fully materialize in situations with inconsistent or weak governance, which would restrict its ability to reduce unemployment (13). As a result, governance conditions rather than enhance artificial intelligence’s role in education, highlighting the significance of a strong legal framework to optimize the technology’s societal impact. This conditional aspect of governance draws attention to a significant vacuum in the literature since most studies have concentrated on how artificial intelligence would directly affect employment rather than thoroughly examining the multifaceted impact of governance on educational attainment.

The study makes several significant contributions to the existing literature by addressing key research gaps and offering a unique theoretical and empirical framework. First, it provides a novel theoretical foundation for understanding the role of education as a mediator and governance quality as a moderator in the relationship between AI and unemployment. The moderated mediation model allows for a deeper understanding of the complex and conditional pathways through which AI impacts unemployment, particularly in high-tech economies. Unlike prior studies that focus solely on the direct impacts of AI on labor markets, this study emphasizes the interplay between technological, educational, and governance factors, offering a more nuanced understanding of AI’s role in fostering inclusive employment. Moreover, a major empirical contribution is using data from 27 high-tech developed countries from 2006 to 2022, incorporating industrial robot installations and AI-related patents as proxies for AI development. This dual measurement approach offers a more comprehensive view of AI’s influence on employment trends. Moreover, the study employs a governance quality index comprising six dimensions—control of corruption, regulatory quality, rule of law, government effectiveness, political stability, and voice and accountability. This enables a detailed analysis of how governance conditions affect the effectiveness of AI in mitigating unemployment for people with disabilities. By focusing on governance, the study highlights the role of strong institutional frameworks in optimizing AI’s societal benefits and addressing systemic challenges in education and employment. Furthermore, a significant contribution of the study is its gender-sensitive analysis of AI’s impact. It reveals that AI-driven automation disproportionately affects men, predominantly employed in automation-prone sectors, while women benefit more from governance policies promoting equitable employment. This gendered perspective adds an important layer to the analysis, emphasizing the need for intersectional approaches in policymaking. Addressing gender disparities in the labor market ensures that AI-driven reforms create equitable opportunities for all. The study also underscores the transformative potential of AI in improving education and skill development. It demonstrates how AI-powered tools like adaptive learning systems and intelligent tutoring can enhance educational outcomes for individuals with disabilities. By reducing physical and cognitive barriers to learning, these tools equip disabled individuals with market-relevant skills, thereby improving their employability in technology-driven economies. Importantly, the study emphasizes that while basic and intermediate education is valuable, advanced education is pivotal in reducing unemployment for disabled individuals. By leveraging AI to enhance advanced education, the study highlights a pathway for disabled individuals to gain specialized skills required for high-demand sectors. From a policy perspective, the study provides actionable recommendations for aligning AI investments with governance and educational reforms. It calls for robust governance frameworks prioritizing inclusivity, transparency, and fairness in AI implementation. Such frameworks are essential for mitigating risks associated with algorithmic biases and ensuring that AI-driven tools are accessible to people with disabilities. Moreover, the study advocates for collaboration between governments, technology firms, and educational institutions to create an ecosystem integrating AI into inclusive educational and labor market systems. By fostering partnerships, policymakers can ensure that technological advancements lead to real improvements in employment outcomes for disabled individuals. Additionally, the study identifies governance quality as a crucial factor in determining AI’s impact. It shows that strong governance amplifies the positive effects of AI on education and employment, while weak governance exacerbates the risks of job displacement caused by automation. This insight highlights the need for tailored governance reforms that maximize the benefits of AI while minimizing its negative consequences. The study’s findings provide a roadmap for designing policies that promote inclusive labor markets and equitable employment opportunities in high-tech economies. To summarize, the study contributes significantly to understanding the mechanisms through which AI impacts unemployment among people with disabilities. Addressing the roles of education and governance provides a comprehensive framework for leveraging AI to foster inclusivity and equity in the labor market. Its theoretical, empirical, and policy insights collectively offer a valuable foundation for future research and practical interventions in the field of AI-driven economic inclusion.

The next sections will present the literature review, empirical strategy, results and discussion, followed by concluding remarks and policy implications.

## 2 Literature review and hypotheses development

### 2.1 Artificial intelligence and unemployment: direct effect

Disabled individuals often experience disproportionately high levels of unemployment and job insecurity, challenges tightly linked to broader socio-economic factors such as poverty, social isolation, and systemic marginalization. These issues are further compounded by limited access to education and training, inflexible work environments, and, in many cases, discriminatory hiring practices that restrict opportunities for meaningful employment. This situation perpetuates cycles of inequality, with long-lasting effects on the lives of disabled individuals and broader economic development. Amid this landscape, artificial intelligence has emerged as a transformative force, with applications that can directly or indirectly address the challenges faced by people with disabilities in accessing and sustaining employment. Artificial intelligence technologies, which span areas such as machine learning, automatization, and adaptive interfaces, are increasingly recognized for their potential to support inclusion in the workforce by enhancing accessibility and skill development, thereby offering a pathway to improve employment outcomes for disabled populations.

Many previous studies support the potential positive impact of AI on employment for disabled individuals by facilitating access to job opportunities through innovative AI-based tools. For instance, Touzet (14) demonstrates that artificial intelligence applications, such as assistive robotics and virtual job-matching platforms, help people with disabilities overcome challenges related to physical and cognitive limitations in the workplace. Touzet’s findings, which align with broader OECD research, show that such technologies enhance employability and sustain long-term employment by adapting tasks to the unique needs of individuals with disabilities. Similarly, the Touzet (14) report emphasizes that AI-driven solutions ranging from assistive technologies to data-driven job-matching systems offer significant opportunities to improve labor market inclusion for people with disabilities, particularly by addressing accessibility barriers and supporting skill development. However, the report also cautions that these benefits depend on overcoming challenges such as algorithmic biases and ensuring equitable access to technology, highlighting the dual nature of AI’s impact. Shuford (15) complements this perspective by finding that artificial intelligence improves accessibility through tools like automated transcriptions and real-time communication aids, which are critical for integrating people with sensory disabilities into collaborative work environments. Together, these studies suggest that while AI holds transformative potential for reducing unemployment among people with disabilities, its effectiveness hinges on careful implementation to mitigate risks and maximize inclusivity.

Other studies explore the benefits of artificial intelligence in facilitating skills training and professional development for disabled individuals, thereby enhancing their job readiness. For example, Man et al. (16) conducted a study on AI-based 3D virtual reality training for individuals with traumatic brain injuries, finding that these AI-driven simulations effectively develop problem-solving and vocational skills. Such AI-enhanced training methods allow individuals with disabilities to practice job-related tasks in a controlled, virtual environment, preparing them for real-world challenges in the workplace. Similarly, Gouvea and Li (17) discuss the role of artificial intelligence in supporting “smart nation” initiatives that focus on using technology to build more inclusive economies. Their global analysis shows that AI-integrated skill development platforms enable individuals with disabilities to acquire competitive skills, enhancing their employability in knowledge-based sectors. In the same vein, Packin (18) underscores that while artificial intelligence offers substantial opportunities for addressing disability employment challenges, careful design is essential to ensuring equitable outcomes. Although Packin’s study primarily focuses on the risks of digital bias, it acknowledges artificial intelligence’s benefits when used to enhance accessibility and inclusivity. By implementing artificial intelligence systems designed to counteract traditional biases and facilitate fair hiring practices, companies can use artificial intelligence to expand employment opportunities for disabled candidates. According to Packin (18), artificial intelligence has the potential to increase transparency and fairness in hiring, thus allowing people with disabilities to compete more equitably in the job market.

Previous studies underscore the role of artificial intelligence as a powerful tool for reducing unemployment among people with disabilities, primarily through enhancing accessibility, supporting personalized education, and enabling adaptive skill development. AI-driven solutions such as assistive devices, adaptive learning platforms, and fair hiring algorithms provide people with disabilities with unprecedented access to job opportunities, making it easier to enter and succeed in the workforce. Studies such as those by Alexiadou (19) and Shuford (15) confirm that artificial intelligence can significantly improve employability by effectively implementing physical and cognitive barriers to employment. Moreover, empirical evidence from Abid et al. (20) and Touzet (14) supports the positive impact of artificial intelligence on improving employment outcomes for disabled individuals. These findings suggest that artificial intelligence has the potential to create a more inclusive labor market, underscoring the importance of continued investment and research in artificial intelligence applications that prioritize accessibility and inclusion. Recent studies, notably Abid et al. (21), have directly explored this impact. In their study, they use a dynamic panel threshold approach to demonstrate that AI reduces the unemployment rate among people with disabilities, particularly in the “upper regime,” that is, for those with secondary and tertiary education levels. This suggests a non-linear relationship, where the benefits of AI become more significant beyond certain thresholds. Therefore, we hypothesize that:

*H1:* AI is expected to reduce unemployment among people with disability.

### 2.2 Mediating role of education

Previous research demonstrates that artificial intelligence can help reduce unemployment among people with disabilities by improving access to education, enhancing its quality, and making it more relevant to labor market needs. The human capital theory provides a framework for understanding the central role of education as a driver of inclusion. According to the literature, education is critical for boosting employability in a labor market transformed by AI advancements (3). Tools such as adaptive learning platforms and intelligent tutoring systems, highlighted in recent studies (14), enable individuals with disabilities to overcome traditional educational barriers and acquire skills aligned with current technological demands. The human capital theory (22) offers a robust theoretical foundation to support this approach: it posits that investment in education increases an individual’s skill set, making them more competitive and adaptable in a changing environment. In this context, AI serves as a catalyst by personalizing learning (4), an advantage that is particularly valuable for people with disabilities, whose needs are often diverse. Education thus emerges as a strategic mechanism through which AI converts technological progress into tangible employment opportunities, thereby reducing unemployment.

Education is a cornerstone of employability, equipping individuals with the skills and qualifications needed to thrive in competitive labor markets, particularly in technology-driven economies. For people with disabilities, conventional education systems often present accessibility barriers that hinder their access to learning and professional development. AI disrupts this dynamic by offering inclusive, adaptive, and personalized educational solutions, essential for enabling them to gain the qualifications demanded in today’s workforce. Studies such as those by Abid et al. (20) show that AI-powered learning platforms provide tailored pathways suited to various profiles, fostering greater participation of individuals with disabilities in educational settings. Through technologies like intelligent tutoring systems, virtual classrooms, and AI-driven learning applications, these individuals benefit from instruction tailored to their pace, abilities, and preferences, creating a truly inclusive educational experience (15). For instance, tools such as text-to-speech software, real-time captioning, and personalized feedback systems assist learners with visual, auditory, or cognitive impairments, making educational content more accessible and allowing them to engage with complex topics independently (14). These innovations do more than remove physical and cognitive barriers; they also pave the way for higher levels of educational attainment, increasing the likelihood of professional integration into high-value sectors where technical and technological skills are in high demand.

The influence of artificial intelligence in educational settings extends beyond accessibility by aligning education with evolving labor market demands, which is essential for enhancing employability among people with disabilities. As the job market increasingly values digital skills and adaptability, AI-driven educational platforms have been instrumental in helping individuals with disabilities acquire relevant, marketable skills. According to Alexiadou (19), artificial intelligence’s role in fostering equitable education lies in its ability to provide targeted training in essential competencies such as coding, data analysis, and digital literacy, which are key skills in high-demand fields. Moreover, artificial intelligence can help bridge the gap between theoretical knowledge and practical skills through simulations and virtual reality environments. Man et al. (16) illustrate this with their study on AI-powered 3D virtual reality vocational training for individuals with traumatic brain injuries, which showed significant improvements in problem-solving and job-specific skills. These training platforms enable people with disabilities to gain hands-on experience, better preparing them for real-world employment. Furthermore, AI-powered career guidance tools and personalized learning pathways help individuals with disabilities navigate their educational and career trajectories, aligning their skills with job market demands and providing pathways to sustainable employment (23). This integration of artificial intelligence into education not only enhances the employability of disabled individuals by making skills training more accessible but also increases their competitiveness in the labor market. By leveraging Artificial intelligence, people with disabilities can overcome traditional educational barriers and gain the qualifications needed to reduce the unemployment gap, thus contributing to a more inclusive economy. We therefore hypothesize:

*H2:* AI is expected to reduce unemployment among people with disabilities by enhancing educational quality.

### 2.3 Moderating effect of governance quality

Institutional theory would shed light on the moderating role of governance as a key determinant of AI’s effectiveness. Our study builds on research showing that governance influences both the efficiency and inclusivity of AI applications (24, 25). Robust governance ensures that AI tools, such as educational platforms or automation systems, are deployed in an ethical and equitable manner (7). According to institutional theory (26), institutions—along with regulatory and policy frameworks, shape economic and social interactions by establishing the “rules of the game. On the one hand, high-quality governance fosters an environment where AI can amplify positive effects on education while moderating its direct impact on employment, for instance, by mitigating algorithmic biases or promoting inclusive policies. On the other hand, governance can paradoxically intensify AI’s negative effects, a point that could be theoretically clarified by noting that poorly aligned institutions may prioritize technological innovation at the expense of social equity. The World Governance Indicators define governance as the customs and establishments that regulate the exercise of power in a nation. This covers how governments are chosen, overseen, and replaced; how well they can create and carry out sensible policies; and how much the public and the government respect the institutions that regulate their social and economic relations (27). Good governance is essential for maximizing the potential of artificial intelligence in enhancing educational quality, as it establishes the regulatory frameworks and ethical standards necessary to ensure that the application of artificial intelligence is equitable, transparent, and effective (24). The literature underscores the importance of governance quality in shaping how artificial intelligence contributes to various sectors, including education, regarding policy support, ethical oversight, and infrastructure (25). When artificial intelligence is deployed in a robust governance environment, its potential to improve educational access and quality is significantly enhanced, enabling educational institutions to adopt AI-driven learning tools that address the diverse needs of students. In the absence of such governance structures, however, the impact of artificial intelligence on education may be hindered, as seen in cases where data privacy issues, algorithmic bias, or lack of regulatory guidance limit artificial intelligence’s inclusivity and effectiveness (8). Effective governance provides the legal and ethical frameworks necessary to address these challenges by mandating that artificial intelligence systems used in education are transparent, safe, and non-discriminatory. In this context, Sharma et al. (25) argue that for artificial intelligence to support effective governance and public services, including education, institutions must establish policies that support innovation while safeguarding public interests. This approach emphasizes the complementing role of governance quality as it encourages artificial intelligence investments that prioritize inclusive and adaptive educational systems, ensuring that educational artificial intelligence applications align with societal values and equity goals. For instance, in contexts where governance frameworks mandate transparency and accountability, the role of artificial intelligence in improving education is more likely to benefit a broader population, fostering an environment where artificial intelligence enhances educational outcomes. For G-7 countries, Saba and Pretorius (24) explore the role of governance as a moderating factor in the relationship between artificial intelligence investment and human well-being within G-7 countries, illustrating how governance can either amplify or restrain artificial intelligence’s benefits depending on the effectiveness of regulatory and policy frameworks. Their findings emphasize that in settings characterized by political stability and transparent institutional governance, artificial intelligence investments are more likely to support improved learning outcomes and human development. This conclusion highlights that governance not only supports the ethical application of artificial intelligence but also mitigates potential negative consequences by ensuring that artificial intelligence deployment aligns with public interest and educational standards. In the same direction, in China, Knox (28) shows that where governance supports artificial intelligence innovation while providing regulatory clarity, AI-driven educational platforms can contribute to skill development at scale, ensuring that education systems remain relevant in an increasingly digital world.

In this context, governance also plays an important role in moderating the impact of AI on some other social issues, such as the unemployment of people with disabilities. Goos and Savona (29) suggest that governance is crucial for society to benefit from AI, ensuring that its deployment does not exacerbate social inequalities. They argue that appropriate governance frameworks can mitigate the risks associated with AI, such as the potential for automation that disproportionately affects vulnerable groups, including people with disabilities. Acemoglu (30) emphasizes that careful management of AI development and its societal impacts is essential to maximize its benefits while minimizing negative effects. He identifies challenges such as excessive worker surveillance and information monopolization as risks that could lead to increased unemployment if governance is not adequate. Saba and Ngepah (31) support this view, highlighting that the impact of AI on employment, particularly in emerging economies such as the BRICS, is strongly moderated by the quality of governance. Effective economic and institutional governance structures can create policies that promote inclusive employment practices and protect against job losses due to AI. However, they also find that political governance with AI can increase unemployment. Furthermore, Leontief (32) also emphasizes the role of technological advancements in economic growth and income distribution, suggesting that without fair governance, technological progress, including AI, can lead to increased inequalities. In the context of business innovation, Lee et al. (33) find that the governance of countries and levels of corruption influence the likelihood of innovation success, indicating that strong governance can foster environments where AI is used to augment rather than replace human labor, thus supporting employment opportunities for people with disabilities. Therefore, good governance is essential not only to improve education sustainably but also to moderate the impact of AI on unemployment, ensuring that its applications are developed and deployed in ways that promote social inclusion and equity. This highlights that the impact of AI on educational quality indeed depends on the strength and adaptability of governance frameworks, which allow AI to contribute positively and equitably to various sectors, including the labor market and education. Hence, we suggest the following hypotheses:

*H3a:* Good governance is expected to strengthen the relationship between AI and educational quality.

*H3b:* Good governance is expected to moderate the relationship between AI and unemployment among people with disabilities.

**Figure 1:**
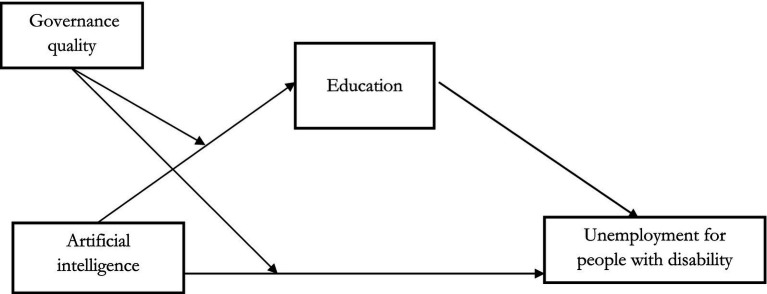
Moderated mediation model for examining AI’s direct and conditional indirect impacts on unemployment among people with disability.

The above discussion shows that when harnessed effectively, artificial intelligence technologies offer the transformative potential to make education more accessible, inclusive, and aligned with labor market demands. However, the extent to which AI-driven educational initiatives positively reduce unemployment depends heavily on governance structures, positioning this relationship within a moderated mediation model. In this model, education serves as a mediating factor through which artificial intelligence reduces unemployment, while governance quality moderates the strength of this mediation. Good governance establishes the regulatory frameworks and ethical guidelines necessary to ensure that artificial intelligence applications in education are equitable, transparent, and effective. For example, in countries with strong governance, policies that support artificial intelligence in education also prioritize inclusivity, ensuring that artificial intelligence systems accommodate diverse learning needs and mitigate potential risks like algorithmic bias. Therefore, as illustrated in Figure 1, the influence of artificial intelligence on reducing unemployment through educational initiatives is contingent on governance quality, shaping how effectively artificial intelligence can be integrated into inclusive educational systems. This moderated mediation framework underscores our assumption that the impact of artificial intelligence on reducing unemployment through educational pathways is optimized under high-quality governance conditions.

*H4:* Governance quality determines the extent to which artificial intelligence reduces unemployment through educational pathways.

## 3 Data and methodology

### 3.1 Data

This study aims to explore the influence of AI on unemployment among individuals with disabilities, focusing on the mechanisms through which this relationship unfolds. In particular, it examines the mediating role of educational level in shaping the impacts of AI on unemployment outcomes among individuals with disabilities. Furthermore, the study considers governance quality as a moderating factor, analyzing how it can amplify or mitigate the impact of AI and education on employment opportunities. The analysis is based on a sample of 27 high-tech developed countries,^1^ over the period 2006–2022. Data sources include international databases such as the World Governance Indicators (WGI), the International Labour Organization, the World Development Indicators. The study period and the sample were determined based on the availability of relevant data. Detailed descriptions of the data sources and variables are presented in Table 1, ensuring transparency and robustness in the empirical analysis. AI serves as the independent variable, while unemployment among people with disabilities is the dependent variable. Educational level, categorized into basic, intermediate, and advanced education, is examined as a mediating variable, while governance quality acts as a moderating factor that may influence the strength of the mediated relationship. Additionally, the analysis includes several control variables, namely government size, foreign direct investment, population growth, economic growth, and inflation to account for other factors that might affect the unemployment outcomes of people with disabilities.

- *Unemployment among people with disabilities* is measured through the unemployment rate with disability. This indicator is sourced from ILOSTAT. It represents individuals who are actively seeking work but are unable to secure employment, categorized based on their disability status. Research, including studies by Abid et al. (2024), uses this data to examine the unemployment challenges faced by individuals with disabilities compared to the broader population.
- *Artificial intelligence*: this study incorporates Artificial intelligence as a key independent variable. Due to its varied applications across numerous sectors, precise evaluation remains complex. Some studies, such as those by Wang et al. (34), use the number of AI-related patents as an indicator, while Li et al. (35) focus on robot application data to examine AI’s impact on resource efficiency. Additionally, researchers such as Duan et al. (36), Wu (37), Yang and Wang (38), and Pi and Fan (39) prioritize data on industrial robot development to assess technological progress. Unlike patents, which often remain at a theoretical stage, industrial robot installation serves as a concrete and accurate measure of AI adoption and maturity. Therefore, in this study, AI development is represented by industrial robot installation data provided by the International Federation of Robotics (40).
- *Education quality* is a mediator variable measured through three complementary dimensions. The first dimension measures the proportion of the labor force that has completed basic education, equivalent to elementary schooling. The second dimension captures the share of the labor force that has attained an intermediate level of education, corresponding to secondary schooling or equivalent. Finally, the third dimension reflects the proportion of the labor force with higher education degrees. These dimensions allow for exploring the differentiated impact of educational qualifications on unemployment access for people with disabilities (41).
- *Governance quality:* In this study, we used governance quality as a moderating variable and applied Principal Component Analysis (PCA) to examine its various indicators by constructing a governance quality index. This index was developed using six governance indicators from Kaufman’s framework, sourced from the World Governance Indicators (WGI). The dimensions include control of corruption, regulatory quality, rule of law, government effectiveness, political stability, and voice and accountability. Each indicator is scored on a scale from −2.5, representing the lowest level of governance, to +2.5, indicating the highest level of governance.

To ensure robustness, we adopted the methodological frameworks proposed by Slimani et al. (59) and Omri et al. (42), who utilized similar indicators in their analyses. By employing PCA, we optimized and grouped the variables, reducing multicollinearity and enhancing the interpretability of the results. In our study, we used Kaiser’s (43) and Jolliffe’s (44) criteria to determine the number of principal components (PCs) to retain from the factor analysis. As recommended by these authors, only PCs with eigenvalues greater than one should be retained. The first PC had an eigenvalue of 4.882 in the general governance model, explaining over 81.37% of the variation in the six underlying variables (see Table 1). The Kaiser-Meyer-Olkin (KMO) and Bartlett’s tests are used to assess the suitability of the data for Principal Component Analysis (PCA). In this case, a KMO of 0.895 indicates excellent suitability, meaning that the variables are strongly correlated with each other and that PCA can be reliably applied. A KMO greater than 0.7 is generally considered a good indicator of the relevance of the analysis. As for Bartlett’s test, with a chi-square value of 3803.447 and a *p*-value of 0.000, rejects the null hypothesis that the variables are independent. This means that the variables are sufficiently correlated to justify the use of PCA. Indeed, the results of both tests confirm that PCA is an appropriate method for these data.

**Table 1:** Principal components analysis.

Components	Eigen value	Proportion	Proportion cumulative
First PC	4.88241	81.37	81.37
Second PC	0.71293	11.88	93.26
Third PC	0.18441	3.07	96.33
Fourth PC	0.12924	2.15	98.48
Fifth PC	0.05029	0.084	99.32
Sixth PC	0.04071	0.006	100
KMO	0.895 3803.447 (0.000)		
Bartlett test			

PC, Principal component.

A set of control variables is incorporated to isolate the impacts of AI on unemployment among individuals with disabilities. These variables are selected based on both the theoretical model and prior research, including inflation rate, economic growth, and government spending (45–48). The annual percentage change in consumer prices quantifies the inflation rate. Existing literature suggests a negative correlation between inflation and unemployment (47). According to the Phillips curve, a stable relationship exists between inflation and unemployment, with rising inflation typically leading to increased employment and reduced unemployment (49). The second control variable, economic growth, is represented by annual GDP growth. Find that economic growth is beneficial in reducing unemployment among people with disabilities. The third control variable, government spending, is represented by the general government’s final consumption expenditure as a percentage of GDP. Recent research presents mixed results: while some studies (50) argue that higher government spending leads to increased unemployment, others (45, 51) suggest it has the opposite effect, helping to reduce unemployment (Table 2).

**Table 2:** Definitions and sources of the variables.

Variables	Signs	Definitions	Sources
Dependents variables			
Unemployment among people with disability	Unmpl	Unemployment rate with disability	ILOSTAT
Independents variables			
Artificial intelligence	AI	Number of AI robots	IFR
Moderator variables			
Governance quality	Gov	Political stability, voice & accountability; rule of law, regulation quality, government effectiveness, control of corruption.	WGI
Mediator variables			
Education levels	Educ	Basic, intermediate and advanced	WDI
Control variables			
Government expediting	EX	General government final consumption expenditure (% of GDP)	WDI
Economic growth	EG	GDP growth (annual %)	WDI
Inflation	Inf	Inflation, consumer prices (annual %)	WDI

ILOSTAT database, International Labour Organization; IFR, International Federation of Robotics; WDI, World Development Indicators.

### 3.2 Research method

Hayes’ macro-process method, developed to analyze complex relationships between variables, is widely used in psychology and social sciences to study mediator and moderator effects (52). It relies on advanced statistical models that explore how intermediate variables (mediators) or contextual factors (moderators) influence causal relationships between independent and dependent variables (53). Compared to alternative techniques such as structural equation modeling (SEM) or hierarchical regression, the Hayes Process Macro offers several advantages, including bias-corrected bootstrapping for indirect effects, improved handling of moderated mediation pathways, and greater interpretability of conditional effects, and an in-depth understanding of the underlying mechanisms of relationships, flexibility in application across various contexts, and greater precision in data analysis through complex models (54). It also allows for controlling confounding effects and is facilitated by tools like the PROCESS macro, which simplifies analysis in software such as SPSS (55).

**Figure 2:**
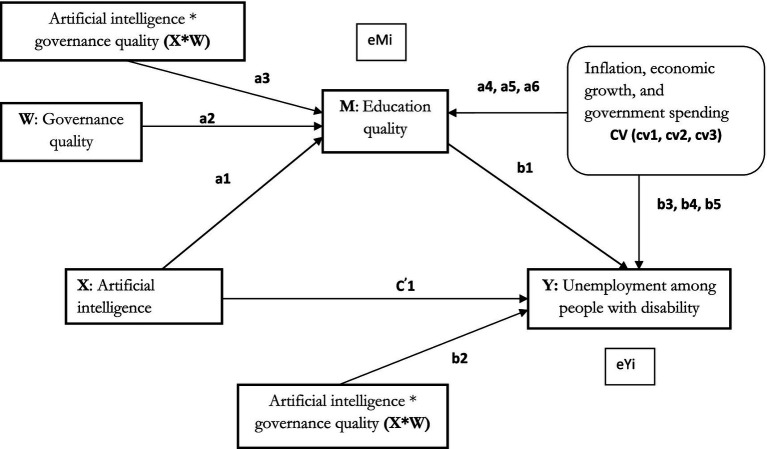
Direct effects of AI, mediating effect of education quality, and conditional moderating effect of governance quality on unemployment among people with disability.

The model outlined in this study assumes a single mediator that plays a causal role between X and Y. Therefore, the analysis is based on the single mediation model (Hayes’ PROCESS 4 model), which specifically evaluates the effect of single mediation. To examine the influence of X on mediator M in the presence of the moderator variable W, the PROCESS 8 model (first-stage moderated mediation model) is employed. As per Hayes (56), a moderated mediation index assesses the relationship between the indirect effect and the moderator variable. Hayes’ (58) first-stage moderation model enables the influence of X on M to be moderated by W within a mediation framework. This process is represented by the Equations 1–6, and as illustrated Figure 2.


(1) $$\text{Direct effect}:\;Y=i_{y}+CX$$

(2) $$\text{Mediation effect}:M=i_{1}+a_{1}X+e_{m}$$

$$Y=i_{2}+C^{\prime }X+b_{1}M+e_{y}$$

Conditional effect: moderated mediation model


(3) $$M=a_{1}+a_{1}X_{1}+a_{2}X+a_{3}XW+e_{m}$$

(4) $$Y=b_{0}+b_{1}M+{C_{1}}^{\prime }X+{C_{2}}^{\prime }W+{C_{3}}^{\prime }XW+e_{y}$$

Calculating indirect and/or conditional effects


(5) $$\text{Direct effect of}\;X\;\text{on}\;Y,\text{conditional}\;\text{on}\;W:{C_{1}}^{\prime }+{C_{3}}^{\prime }W$$

(6) $$\text{Indirect effect of}\;X\;\text{on}\;Y,\text{conditional}\;\text{on}\;W:\left(a_{1}+a_{3}W\right)b_{1}$$

Hayes (57) proposes a method for evaluating the influence of the moderator variable W on the indirect effect of X on Y through the mediator M within the framework of a first-stage moderated mediation model. In this model, the coefficient ab quantifies the degree to which W moderates the indirect effect, effectively serving as a key indicator of moderate mediation. To ensure the reliability of this index, Hayes (57) advocates for using a bias-corrected bootstrap confidence interval calculated from 5,000 bootstrap samples. This bootstrapping technique allows for a more accurate estimation of the moderated mediation effect by accounting for sampling variability and minimizing potential biases. By generating multiple resamples, the method provides a robust confidence interval that reflects the true relationship between the indirect effect and the moderator variable, enhancing the precision and validity of the moderation analysis. This approach is especially valuable in cases where the distribution of the indirect effect may not be normal, as it does not rely on parametric assumptions, making it a powerful tool for testing the significance of moderated mediation effects.

## 4 Results and discussion

### 4.1 Descriptive analysis

Descriptive statistics for high-tech developed countries from 2006 to 2022, as shown in Table 3, reveal a high unemployment rate among individuals with disabilities. The average rate is 0.6273, with a standard deviation of 0.4562 and a range from 0.0295 to 5.04. This demonstrates significant variation across countries, with some reporting very low rates while others show much higher levels. Regarding AI adoption, although the average is relatively high at 4.0259, the standard deviation is 1.875, and the values range from 0.6931 to 11.981, reflecting a large disparity between countries, with some being very advanced while others lag significantly behind. As for the mediator variables, basic and intermediate education levels are quite homogeneous, with low variation: the average for basic education is 3.6017 (standard deviation of 0.3526), with values ranging from 2.6446 to 4.3631, and for intermediate education, the average is 4.2052 (standard deviation of 0.0843), with a very narrow range of 4.015 to 4.4195, suggesting relatively equal access to education in these countries. However, advanced education shows more disparities, with an average of 2.1830, a standard deviation of 0.7934, and values ranging from 1.8240 to 4.500, indicating significant gaps between countries regarding access to higher education. The quality of governance is considered a moderating variable in the study, with an average of 1.20, a standard deviation of 0.4566, and values ranging from 0.155 to 1.9782. This variation reflects notable differences in public management and government transparency. As for the control variables, there are also significant variations, with an average GDP growth of 1.9318, a standard deviation of 3.9795, and values ranging from −14.838 to 24.475, showing large economic fluctuations between countries, with some experiencing difficult periods while others enjoy sustained growth. Public spending, with an average of 19.9638 (standard deviation of 3.4311), varies between 10.424 and 27.935, indicating significantly different public spending priorities across countries. Finally, inflation, with an average of 2.3274, a standard deviation of 2.8013, and values ranging from −4.447 to 19.705, shows considerable instability, with some countries experiencing periods of deflation while others face high inflation rates.

**Table 3:** Preliminary analysis.

Variable	Obs	Mean	Std.	Min	Max
Unemp	423	0.6273883	0.456253	0.0295519	5.044943
AI	441	4.025962	1.875125	0.6931472	11.98687
Basic	442	3.601759	0.3526233	2.644613	4.363175
Inter	442	4.205244	0.0834662	4.015914	4.419587
Advan	437	2.183043	0.7934773	1.824015	4.500554
Gov	442	1.200437	0.4566544	0.1556425	1.978271
GDP	442	1.931861	3.979547	−14.83861	24.47525
EXP	442	19.96383	3.431176	10.42441	27.935
Infl	442	2.327427	2.801307	−4.447547	19.70505

Unemp, Unemployment among people with disability, AI, artificial intelligence; Basic, Basic education; Inter, Intermediate education; Advan, Advanced education; Gov, Index of governance quality; GDP, Economic growth; EXP, Gouverment expenditure.

**Figure 3:**
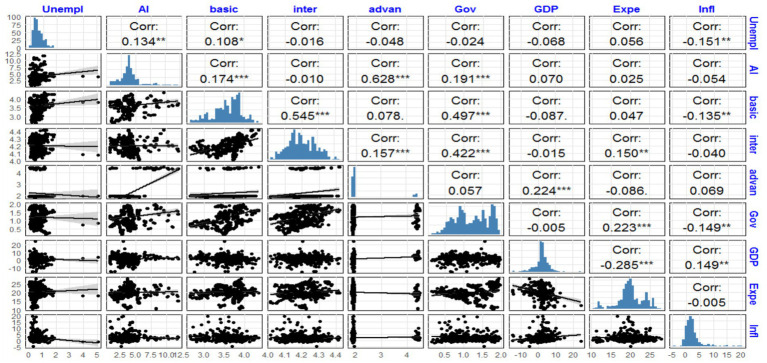
Scatter plot matrix, histogram, and correlation matrix of variables.

The scatterplot matrix, histograms, and correlation matrix in Figure 3 visually and statistically represent the relationships between the variables studied. First, the correlation matrix reveals that the unemployment rate is positively correlated with AI adoption (correlation of 0.134, significant at 5%), suggesting a potential increase in unemployment among disabled individuals with higher levels of AI adoption. Conversely, unemployment is negatively correlated with inflation (correlation of −0.151, significant at 5%), which may reflect the complex dynamics between economic instability and employment among vulnerable populations. AI adoption also shows notable correlations with education levels: it is strongly correlated with intermediate education (correlation of 0.545, significant at 1%) and more moderately with advanced education (correlation of 0.157). Additionally, a positive relationship exists between AI adoption and governance quality (correlation of 0.191, significant at 1%), indicating that countries with better governance are more likely to adopt advanced technologies. Furthermore, governance quality is positively correlated with GDP growth (correlation of 0.224, significant at 1%), suggesting that better governance practices promote greater economic growth. The histograms confirm that the distributions of variables are uneven. For instance, the unemployment rate is skewed, with most countries having low rates but a few exhibiting high values. AI adoption is also skewed, with certain countries emerging as leaders in the field. Intermediate education is concentrated around the mean, whereas advanced education shows greater variability. Finally, the scatterplots confirm the identified correlations. These observations highlight the complex relationships between governance, education, AI adoption, and economic indicators while underscoring significant disparities among countries.

### 4.2 Conditional process analysis

#### 4.2.1 Direct and indirect impact of AI on unemployment among people with disability

The findings from Tables 4–6 show that the direct effect of AI on the unemployment of disabled individuals is significant but positive in all cases. For example, for basic education (Table 4), the direct effect of AI is 0.0304 (*p* = 0.0024, CI [0.0109; 0.0499]). Similarly, for advanced education (Table 6), the direct effect is 0.0662 (*p* < 0.0001, CI [0.0395; 0.0928]). These results suggest that AI does not reduce unemployment among disabled individuals. On the contrary, it exacerbates disparities, particularly due to task automation and inadequate education and vocational training systems. These findings contradict hypothesis H1, which assumed that artificial intelligence would directly reduce unemployment among disabled individuals. Several factors can explain this increase. First, task automation tends to eliminate jobs that were traditionally accessible to disabled people. Second, emerging technologies are still insufficiently adapted to the specific needs of this population, creating an additional barrier to their integration. Finally, the gap between the current skills of disabled individuals and the demands of a labor market dominated by AI exacerbates employment access difficulties. These conclusions, supported by studies such as those by Qin et al. (48), show that AI could paradoxically increase unemployment among disabled individuals due to the increased automation of certain tasks and the slow adaptation of technologies to the specific needs of these populations. Qin et al. (48) emphasize the importance of inclusivity in implementing technologies, suggesting that AI could exacerbate existing inequalities, especially if educational and vocational training systems are not adapted to the needs of disabled people. This phenomenon is also discussed by Nguyen and Vo (47), who argue that, internationally, AI and automation can have negative effects on employment, particularly for vulnerable groups who do not benefit from tailored training. Pi and Fan (40) add to this reflection by studying the impact of robots on unemployment, noting that automation in specific sectors could lead to job reductions for already marginalized workers, particularly in unionized environments where disabled workers are less represented. Finally, Bordot’s (58) work on the impact of robots and AI in OECD countries highlights that introducing these technologies can initially lead to an increase in unemployment, especially if governments and businesses do not take proactive measures to support the professional transitions of disabled individuals. These results suggest that AI, without public policies and tailored training, can intensify disparities rather than reduce them.

**Table 4:** Direct and indirect impacts of AI on unemployment for people with disabilities through Basic Education.

	M (Basic education)					Y (Unemployment among people with disability)				
Variables	Coeff		SE (HC4)	P	IC 95%	Coeff		SE (HC4)	P	IC 95%
Artificial intelligence	a1	0.0394	0.0095	0.0000	[0.0208; 0.0580]	c’1	0.0304	0.0099	0.0024	[0.0109; 0.0499]
Basic education						b1	0.0776	0.0541	0.1517	[−0.0286; 0.1839]
GDP	a4	−0.0091	0.0048	0.0561	[−0.0185; 0.0002]	b4	−0.005	0.0047	0.2886	[−0.0143; 0.0043]
Government expenditure	a5	0.0025	0.0041	0.5452	[−0.0055; 0.0105]	b5	0.0047	0.0052	0.3732	[−0.0056; 0.0150]
Inflation	a6	−0.014	0.0057	0.0134	[−0.0252; −0.0029]	b6	−0.0206	0.007	0.0034	[−0.0343; −0.0069]
Constant	i1	3.4314	0.0991	0.0000	[3.2366; 3.6261]	i2	0.1902	0.2034	0.3503	[−0.2096; 0.5899]
*R*	0.5559					0.5152				
*R* ^2^	0.6550					0.4063				
F (HC4)	27.0903 (0.000)					12.9762 (0.000)				

Direct and indirect effect of AI on Unemployment among people with disability				
	Coeff	SE (HC4)	P	IC 95% (LLCI; ULCI)
Total effect	0.0334	0.0096	0.0005	[0.0146; 0.0523]
Direct effect	0.0304	0.0099	0.0024	[0.0109; 0.0499]
Indirect effect	0.0031	0.0024	–	[−0.0011; 0.0083]

SE, Standard error; IC, Interval of confidence; P, Plus-value.

**Table 5:** Direct and indirect impacts of AI on unemployment for people with disabilities through intermediate education.

	M (intermediate education)					Y (Unemployment among people with disability)				
Variables	Coeff		SE (HC4)	P	IC 95%	Coeff		SE (HC4)	P	IC 95%
Artificial intelligence	a1	0.0000	0.0023	0.9958	[−0.0046, 0.0046]	c’1	0.0334	0.0096	0.0005	[0.0146, 0.0522]
Intermediate education						b1	−0.1713	0.3358	0.6102	[−0.8314, 0.4888]
GDP	a4	0.0002	0.0011	0.8244	[−0.0019, 0.0024]	b4	−0.0057	0.0046	0.2220	[−0.0148, 0.0035]
Government expenditure	a5	0.0039	0.0012	0.0008	[0.0016, 0.0062]	b5	0.0055	0.0048	0.2503	[−0.0039, 0.0150]
Inflation	a6	−0.0014	0.0014	0.3319	[−0.0042, 0.0014]	b6	−0.0219	0.0075	0.0035	[−0.0366, −0.0072]
Constant	i1	4.1281	0.0240	0.0000	[4.0809, 4.1753]	i2	1.1638	1.4386	0.4190	[−1.6641, 3.9917]
*R*	0.3012					0.4365				
*R* ^2^	0.4412					0.5321				
F (HC4)	9.0215 (0.000)					11.268 (0.000)				

Direct and indirect effect of AI on Unemployment among people with disability				
	Coeff	SE (HC4)	P	IC 95% (LLCI; ULCI)
Total effect	0.0334	0.0096	0.0005	[0.0146, 0.0523]
Direct effect	0.0334	0.0096	0.0005	[0.0146, 0.0522]
Indirect effect	0.0000	0.0009	–	[−0.0018, 0.0019]

SE, Standard error; IC, Interval of confidence; P, Plus-value.

**Table 6:** Direct and indirect impacts of AI on unemployment for disabled people via advanced education.

	M (Advanced education)					Y (Unemployment among people with disability)				
Variables	Coeff		se (HC4)	P	IC95%	Coeff		se (HC4)	P	IC 95%
Artificial intelligence	a1	0.2711	0.0239	0.000	[0.2241; 0.3181]	c’1	0.0662	0.0136	0.000	[0.0395; 0.0928]
Advanced education						b1	−0.1187	0.0341	0.0005	[−0.1857; −0.0517]
GDP	a4	0.0277	0.0104	0.0079	[0.0073; 0.0480]	b4	−0.0026	0.0049	0.5978	[−0.0121; 0.0070]
Government expenditure	a5	−0.013	0.0066	0.0499	[−0.0259; 0.0000]	b5	0.0032	0.0053	0.544	[−0.0072; 0.0136]
Inflation	a6	0.0234	0.0111	0.0365	[0.0015; 0.0453]	b6	−0.0187	0.0071	0.0085	[−0.0326; −0.0048]
Constant	i1	1.2168	0.1658	0.000	[0.8909; 1.5426]	i2	0.6003	0.1348	0.000	[0.3354; 0.8652]
*R*	0.6710					0.5525				
*R* ^2^	0.4502					0.637				
F (HC4)	44.4786 (0.000)					25.0268 (0.000)				

Direct and indirect effect of AI on unemployment				
	Effect	se (HC4)	P	(IC) 95%
Total effect	0.034	0.0097	0.0005	[0.0149; 0.0530]
Direct effect	0.0662	0.0136	0.0000	[0.0395; 0.0928]
Indirect effect	−0.0322	0.0102	–	[−0.0550; −0.0151]

SE, Standard error; IC, Interval of confidence; P, Plus-value.

**Figure 4:**
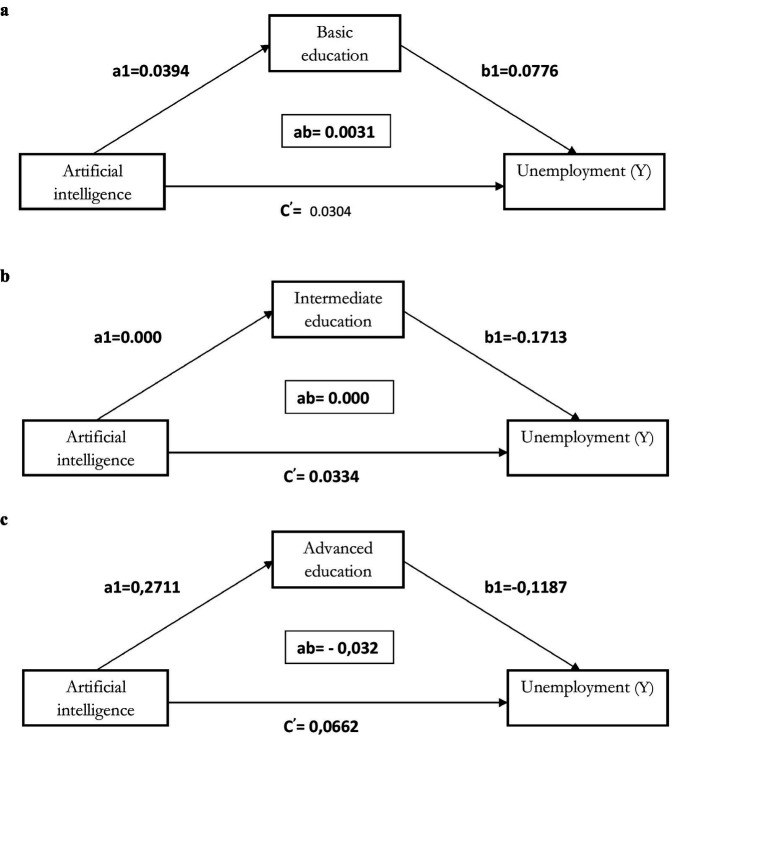
**(a)** Simple mediation model for Artificial intelligence - unemployment for people with disability with basic education as mediator. **(b)** Simple mediation model for Artificial intelligence - unemployment for people with disability with intermediate education as mediator. **(c)** Simple mediation model for Artificial intelligence - unemployment for people with disability with advanced education as mediator.

The analysis of the indirect effects of AI through education, as shown in the tables, reveals significant differences based on education levels. As shown in Figure 4a and detailed in Table 4, the indirect effect of AI via basic education on the unemployment of disabled individuals is weak and not significant. The coefficient associated with this indirect effect is Coeff = 0.0031 with a confidence interval of [−0.0011; 0.0083], indicating no clear relationship between AI, basic education, and unemployment. The effect of AI on basic education, represented by the coefficient a1, is slightly positive but weak (a1 = 0.0152). Moreover, the effect of basic education on unemployment, represented by the coefficient b1, is also weak and insignificant (b1 = 0.2039, CI = [−0.1408; 0.5486]). These results suggest that while AI may have a marginal effect on improving basic education, it does not seem sufficiently adapted to meet the technological demands of the labor market, especially regarding disabled individuals. In other words, the skills acquired at the basic education level are not robust enough to face the challenges of the digital economy.

In contrast, as shown in Figure 4b and clarified in Table 5, the indirect effect of AI via intermediate education is also weak and not significant. The coefficient associated with this indirect effect is Coeff = 0.0000, with a confidence interval of [−0.0018; 0.0019], indicating that intermediate education does not contribute significantly to reducing unemployment among disabled individuals in the context of AI. The effect of AI on intermediate education, represented by coefficient a1, is very close to zero (a1 = 0.0031), suggesting that AI does not significantly impact this level of education. Similarly, the effect of intermediate education on unemployment, represented by the coefficient b1, remains weak and insignificant (b1 = 0.1823, CI = [−0.2087; 0.5733]). This shows that even though intermediate education may be useful for disabled individuals, it does not seem to play a major role in combating unemployment, especially in an environment where digital skills are increasingly required. Finally, as shown in Figure 4c and detailed in Table 6, the indirect effect of AI via advanced education on the unemployment of disabled individuals is significant and negative. The coefficient associated with this indirect effect is Coeff = −0.0322, with a confidence interval of [−0.0550; −0.0151], indicating a significant relationship. The effect of AI on advanced education, represented by the coefficient a1, is positive and significant (a1 = 0.0487), showing that AI can help improve access to advanced education for disabled individuals. Moreover, the effect of advanced education on unemployment, represented by the coefficient b1, is negative and significant (b1 = −0.6610, CI = [−0.9852; −0.3368]). This means that advanced education is a key factor in reducing unemployment, particularly in a technological environment where specialized skills are required. Individuals with access to advanced education appear better prepared to meet the challenges of the labor market and take advantage of opportunities offered by AI.

In summary, the indirect effects of AI through basic, intermediate, and advanced education reveal contrasting results. While basic education, slightly improved by AI, does not play a determining role in reducing unemployment among disabled individuals, intermediate education also does not provide a notable contribution in this area. In contrast, advanced education, supported by AI, stands out as a significant lever for reducing unemployment and improving the employability of disabled individuals. These results highlight the need to strengthen access to specialized training tailored to the needs of the digital labor market, especially for disabled individuals. They partially confirm hypothesis H2, which postulates that AI reduces unemployment among disabled individuals by improving the quality of education. This reduction is primarily observed through advanced education, which enables the acquisition of essential skills to meet the growing demands of a technological environment. These results corroborate previous studies, highlighting that the quality of education, particularly advanced education, allows individuals to acquire specialized skills essential in a labor market increasingly oriented toward innovation and technology (19). Key skills, such as proficiency in digital tools, programming, or data analysis, are crucial to remain competitive in the modern economy. AI’s positive and significant impact on access to advanced education is particularly revealing. AI-based learning technologies, such as intelligent tutoring platforms or virtual learning environments, allow disabled individuals to benefit from specialized training, overcoming physical or cognitive barriers often present in traditional educational systems (15). This increased accessibility is especially valuable in a context where AI and digital technologies are key drivers of economic growth. By facilitating access to advanced education for disabled individuals, AI plays a central role in improving their employability and reducing unemployment gaps. Furthermore, advanced education’s negative and significant effect on unemployment confirms its key role in adapting to the demands of a constantly evolving labor market. This observation highlights the importance of developing specialized skills, particularly in rapidly expanding sectors such as artificial intelligence, big data, and cybersecurity (23).

#### 4.2.2 Conditional effect of AI on education quality

Table 7 presents the results of the analysis of the effects of AI, governance, and their interaction on the quality of education at three levels: basic, intermediate, and advanced education. These results highlight significant differences depending on the level of education and emphasize the moderating role of governance. For the direct effect of AI, the results show that it has a positive and significant impact on the quality of basic education (Coeff = 0.0248, *p* = 0.0036) and advanced education (Coeff = 0.2768, *p* = 0.0000). This indicates that AI improves the quality of education at these levels. However, no significant direct effect is observed for intermediate education (Coeff = −0.0029, *p* = 0.2425), suggesting that AI does not have a measurable impact at this level. Regarding governance, it has a significant direct effect on basic education (Coeff = 0.3826, *p* = 0.0000) and intermediate education (Coeff = 0.0793, *p* = 0.0000), showing that strong governance improves the quality at these levels. In contrast, its direct effect is not significant for advanced education (Coeff = −0.1033, *p* = 0.0979), indicating that governance does not directly influence this level of education.

**Table 7:** Conditional effect of AI on education quality (Basic, intermediate and advanced).

	M (Basic education)					M (intermediate education)				M (advanced education)			
Variables		Coeff.	SE (HC4)	P	IC 95%	Coeff.	SE (HC4)	P	IC 95%	Coeff.	SE (HC4)	P	IC 95%
AI (X)	a1	0.0248	0.0085	0.0036	[0.0082; 0.0415]	−0.0029	0.0025	0.2425	[−0.0079; 0.0020]	0.2768	0.0257	0.0000	[0.2263; 0.3272]
Governance (W)	a2	0.3826	0.0299	0.000	[0.3239; 0.4412]	0.0793	0.0067	0.0000	[0.0662; 0.0924]	−0.1033	0.0623	0.0979	[−0.2257; 0.0191]
Interaction (W*X)	a3	−0.0119	0.019	0.531	[−0.0493; 0.0255]	0.0086	0.0061	0.1549	[−0.0033; 0.0205]	0.2205	0.0649	0.0007	[0.0929; 0.3480]
GDP	a4	−0.0131	0.0044	0.0029à	[−0.0217; −0.0045]	−0.0005	0.0010	0.6427	[−0.0024; 0.0015]	0.0305	0.0094	0.0014	[0.0119; 0.0490]
Government expenditure	a5	−0.0099	0.0027	0.0003	[−0.0153; −0.0045]	0.0014	0.0011	0.2062	[−0.0008; 0.0037]	−0.0077	0.0062	0.2176	[−0.0199; 0.0045]
Inflation	a6	−0.0047	0.0056	0.4039	[−0.0157; 0.0064]	0.0004	0.0014	0.7546	[−0.0023; 0.0032]	0.0186	0.0098	0.0591	[−0.0007; 0.0379]
Constant	i1	3.8247	0.0587	0.000	[3.7094; 3.9400]	4.1732	0.0237	0.0000	[4.1266; 4.2199]	2.173	0.1334	0.0000	[1.9108; 2.4351]
*R*	0.5331					0.5468				0.7078			
*R* ^2^	0.3842					0.5901				0.5010			
F (HC4)	32.9023 (0.000)					26.6422 (0.000)				46.3905 (0.000)			

Test(s) of highest order unconditional interactions (Moderating effect of governance)															
	M (Basic education)					M (intermediate education)					M (advanced education)				
	R2-chng	F(HC4)	df1	df2	P	R2-chng	F(HC4)	df1	df2	P	R2-chng	F(HC4)	df1	df2	P
Interaction (X*W)	0.0006	0.3931	1	415	0.5310	0.0058	2.0303	1	415	0.1549	0.0466	11.5424	1	410	0.0007

Conditional effect at different values of governance													
		M (Basic education)				M (intermediate education)				M (advanced education)			
	Gov	Effect	SE (HC4)	P	IC 95%	Effect	SE (HC4)	P	IC 95%	Effect	SE (HC4)	P	IC 95%
Low	−0.5231	–	–	–	–	–	–	–	–	0.1599	0.0531	0.0028	[0.0555; 0.2644]
Middle	0.0251	–	–	–	–	–	–	–	–	0.2823	0.0248	0.0000	[0.2336; 0.3310]
High	0.5460	–	–	–	–	–	–	–	–	0.3971	0.03	0.0000	[0.3381; 0.4561]

SE, Standard error; IC, Interval of confidence; P, Plus-value.; Lower = 16th percentile; Middle: 50th percentile; Upper: 84th percentile.

**Figure 5:**
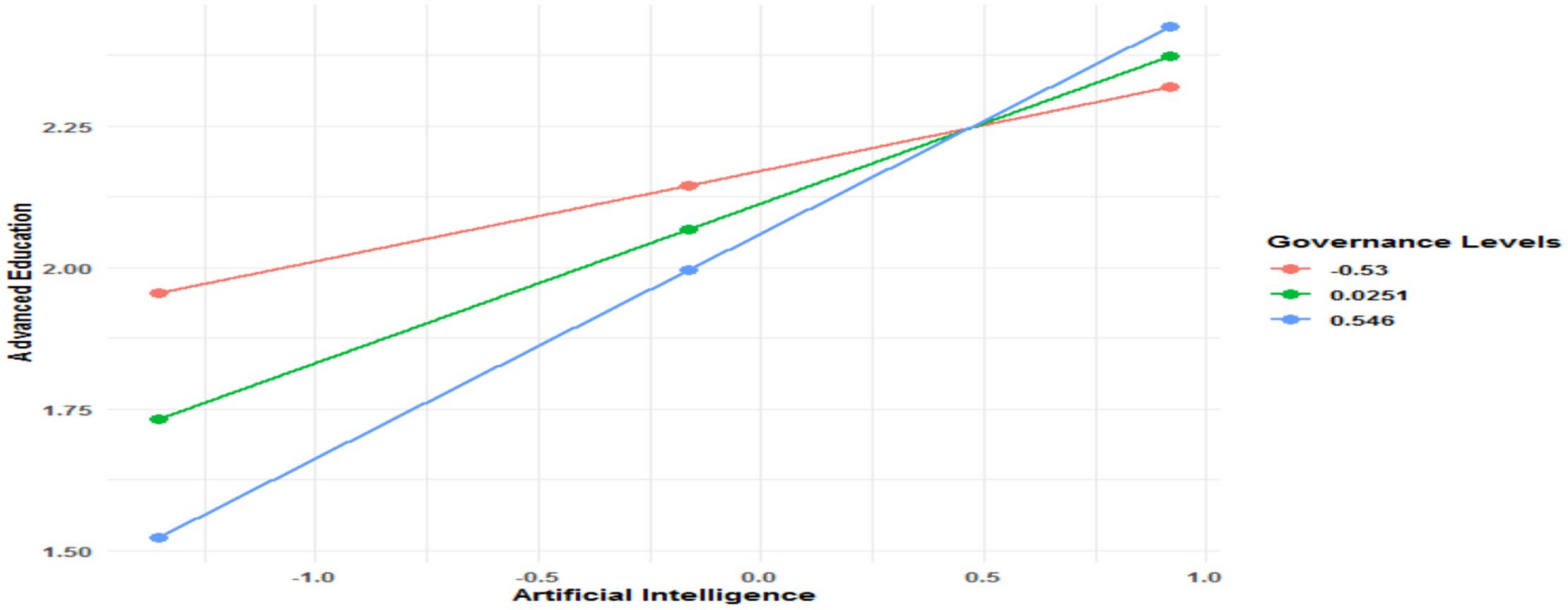
Conditional effect of artificial intelligence on advanced education in the presence of governance quality.

Moreover, the interaction between AI and governance reveals interesting results. For basic and intermediate education, the interaction is insignificant (*p* = 0.5310 and *p* = 0.1549, respectively), meaning that governance does not modify the effect of AI on these levels. However, for advanced education, the interaction is significant and positive (Coeff = 0.2205, *p* = 0.0007), showing that governance strengthens the effect of AI. Thus, effective governance amplifies the benefits of AI for this level of education. Conditional interaction tests based on governance levels confirm these observations. The moderating effect of governance is not significant (*R*^2^-chng = 0.0006, *p* = 0.5310 for basic education and *R*^2^-chng = 0.0058, *p* = 0.1549 for intermediate education). In contrast, for advanced education, the moderating effect is significant and substantial (*R*^2^-chng = 0.0466, *p* = 0.0007), indicating that governance plays a key role in amplifying the effect of AI. In other words, the impact of AI on advanced education depends on the level of governance. Figure 5 clearly illustrates the conditional effect of AI on advanced education, moderated by the level of governance. The visual results confirm the analysis of the conditional effects presented in Table 7, showing that the impact of AI on advanced education increases systematically with higher levels of governance. Specifically, when governance is low, the effect is limited (Effect = 0.1599, *p* = 0.0028). This effect becomes more pronounced at a medium level of governance (Effect = 0.2823, *p* = 0.0000) and reaches its maximum in environments with high governance (Effect = 0.3971, *p* = 0.0000). These results highlight the importance of strong governance in maximizing the benefits of AI in advanced education.

Hypothesis H3.1, which suggests that good governance strengthens the relationship between AI and the quality of education, is partially confirmed by the results. For basic and intermediate education, governance does not play a significant moderating role, indicating that its influence on the effect of AI at these levels is negligible. In contrast, for advanced education, governance acts as a key moderator, amplifying the positive impact of AI on the quality of education. These results highlight that governance is particularly essential in more complex educational contexts, where its role helps maximize the benefits of AI. This suggests that the impact of governance is contextual and more relevant for education levels requiring more advanced resources and skills. This study’s findings corroborate previous studies’ conclusions, confirming the idea that the impact of governance is contextual and depends on the specific requirements of each educational level. Indeed, as Saba and Pretorius (24) highlight, effective governance establishes the necessary standards to ensure that the use of AI in education is equitable, transparent, and inclusive. This could explain why the moderating effect of governance is significant in the context of advanced education, where the needs are more specific and require substantial resources. For example, in these contexts, governance can play a crucial role in regulating AI-based learning tools, ensuring their safety, reliability, and relevance to meet the needs of a diverse student population. Moreover, according to Sharma et al. (25), quality governance encourages innovation while safeguarding the public interest. In advanced education, this translates into policies that promote the integration of adaptive and inclusive AI-based learning tools that can meet the specific needs of a wide range of learners. Thus, strong institutional frameworks enable more effective adoption of AI and strengthen its positive impact on the quality of education.

#### 4.2.3 Conditional direct impacts of AI on unemployment among people with disability

**Figure 6:**
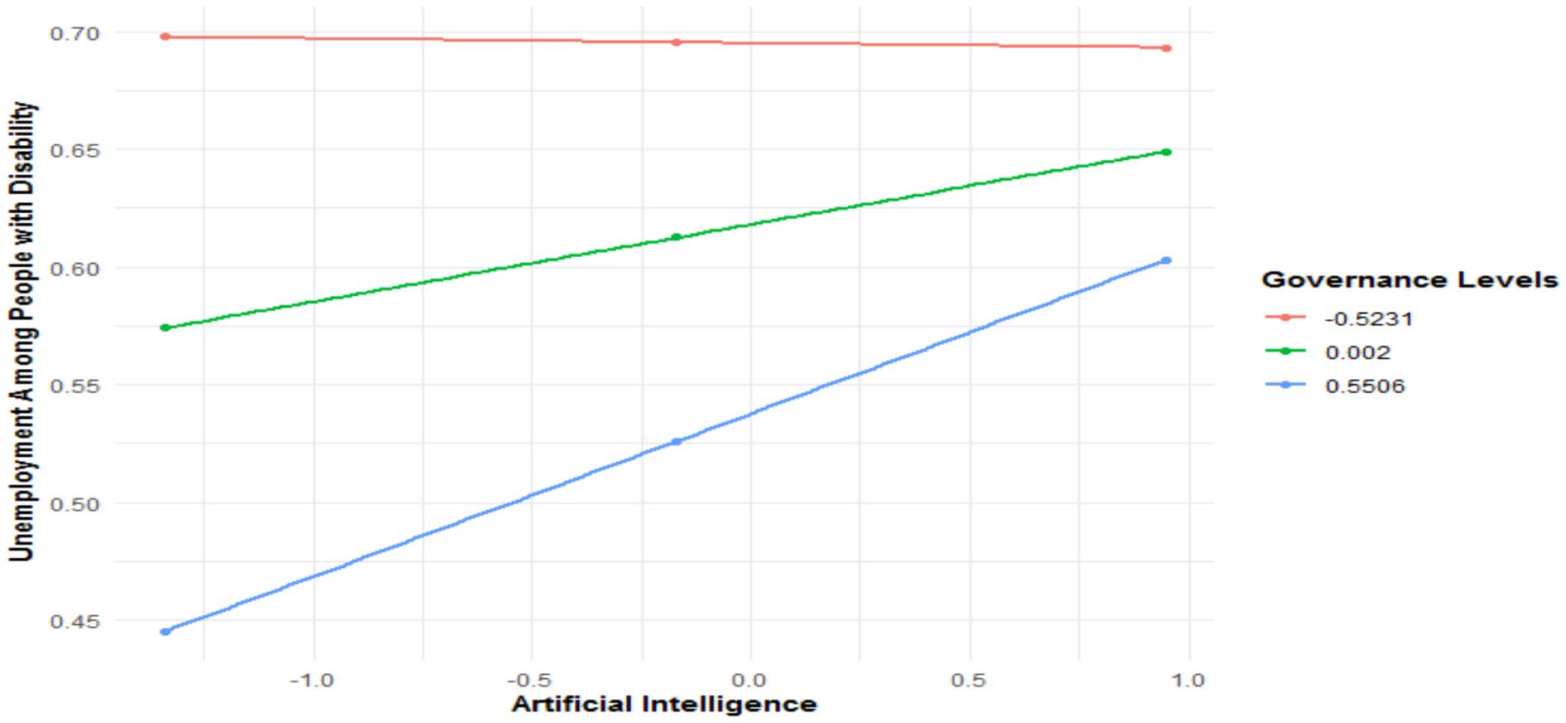
Conditional effect of AI on unemployment among people with disability in the presence of governance quality.

Table 8 highlights the conditional impacts of AI on the unemployment of people with disabilities, moderated by governance, across three models corresponding to education levels: basic, intermediate, and advanced. In the model corresponding to the basic education level, the direct effect of AI on unemployment is weak but significant, with a coefficient of 0.0327 (*p* = 0.0012). This reveals a slight increase in unemployment for individuals with a low level of education due to the introduction of AI. Meanwhile, governance significantly negatively affects unemployment (−0.1465, *p* = 0.0223), underscoring its overall role in reducing unemployment. However, the interaction between AI and governance (X*W) shows a positive coefficient of 0.0662 (*p* = 0.011), indicating that governance paradoxically amplifies the negative effect of AI on unemployment. The interaction test (*R*^2^-chng = 0.0114, F(HC4) = 6.5270, *p* = 0.0110) confirms the significance of this interaction. As shown in Figure 6, the conditional effects reveal that with low governance, the effect of AI on unemployment is 0.0308 (*p* = 0.0398). For medium governance, this effect increases to 0.0464 (*p* = 0.0082). Finally, under high governance, the effect reaches 0.0619 (*p* = 0.0021). These results suggest that while governance contributes overall to reducing unemployment, it simultaneously exacerbates the negative effects of AI in this context.

**Table 8:** Conditional impact of AI on unemployment among people with disability.

Y (Unemployment among people with disability)													
	M (Basic education)					M (intermediate education)				M (advanced education)			
Variables		Coeff.	SE (HC4)	P	IC 95%	Coeff.	SE (HC4)	P	IC 95%	Coeff.	SE (HC4)	P	IC 95%
AI (X)	c’1	0.0327	0.01	0.0012	[0.0130; 0.0523]	0.0368	0.0097	0.0002	[0.0178; 0.0558]	0.083	0.0137	0.0000	[0.0560; 0.1099]
Basic		0.1717	0.0741	0.021	[0.0260; 0.3174]	–	–	–	–	–	–	–	–
Inter	b1	–	–	–	–	−0.0430	0.3256	0.8950	[−0.6830; 0.5970]	–	–	–	–
Advanced		–	–	–	–	–	–	–	–	−0.1638	0.0348	0.0000	[−0.2322; −0.0954]
Governance (W)	b2	−0.1465	0.0639	0.0223	[−0.2720; −0.0210]	−0.0774	0.0408	0.0584	[−0.1576; 0.0028]	−0.0977	0.0483	0.0436	[−0.1926; −0.0028]
Interaction (W*X)	b3	0.0662	0.0259	0.011	[0.0153; 0.1171]	0.0645	0.0262	0.0142	[0.0130; 0.1160]	0.0994	0.0231	0.0000	[0.0540; 0.1449]
GDP	b4	−0.002	0.0053	0.7039	[−0.0124; 0.0084]	−0.0043	0.0048	0.3748	[−0.0137; 0.0052]	0.0005	0.0051	0.9152	[−0.0094; 0.0105]
Government expenditure	b5	0.0097	0.0048	0.0434	[0.0003; 0.0192]	0.0081	0.0047	0.0862	[−0.0012; 0.0173]	0.0066	0.0048	0.1712	[−0.0029; 0.0161]
Inflation	b6	−0.0235	0.0077	0.0026	[−0.0387; −0.0082]	−0.0242	0.0079	0.0022	[−0.0397; −0.0088]	−0.021	0.0077	0.0068	[−0.0362; −0.0058]
Constant	i2	−0.135	0.2791	0.6288	[−0.6835; 0.4135]	0.7013	1.3879	0.6136	[−2.0269; 3.4294]	0.8798	0.1457	0.0000	[0.5934; 1.1662]
*R*	0 0.5693					0.4245				0.6087			
*R* ^2^	0.4552					0.5981				0.7253			
F (HC4)	10.8163 (0.000)					9.8170 (0.000)				27.9968 (0.000)			

Test(s) of highest-order unconditional interactions (Moderating effect of governance)															
	M (Basic education)					M (intermediate education)					M (advanced education)				
	R2-chng	F(HC4)	df1	df2	P	R2-chng	F(HC4)	df1	df2	P	R2-chng	F(HC4)	df1	df2	P
Interaction (X*W)	0.0114	6.5270	1	414	0.0110	0.0107	6.0683	1	414	0.0142	0.0466	11.5424	1	410	0.0007

Conditional direct effect at different values of governance													
		M (Basic education)				M (intermediate education)				M (advanced education)			
	Governance	Effect	SE (HC4)	P	IC 95%	Effect	SE (HC4)	P	IC 95%	Effect	SE (HC4)	P	IC 95%
Low	−0.5231	−0.0019	0.0126	0.8777	[−0.0267; 0.0228]	0.0031	0.0124	0.8038	[−0.0213; 0.0274]	0.0303	0.0146	0.0392	[0.0015; 0.0590]
Middle	0.0251	0.0328	0.01	0.0011	[0.0131; 0.0525]	0.037	0.0097	0.0002	[0.0179; 0.056]	0.0855	0.0139	0.0000	[0.0581; 0.1129]
High	0.5460	0.0691	0.0209	0.001	[0.0281; 0.1101]	0.0723	0.0209	0.0006	[0.0313; 0.1134]	0.1372	0.0218	0.0000	[0.0944; 0.1801]

SE, Standard error; IC, Interval of confidence; P, Plus-value.; Lower = 16th percentile; Middle: 50th percentile; Upper: 84th percentile.

**Figure 7:**
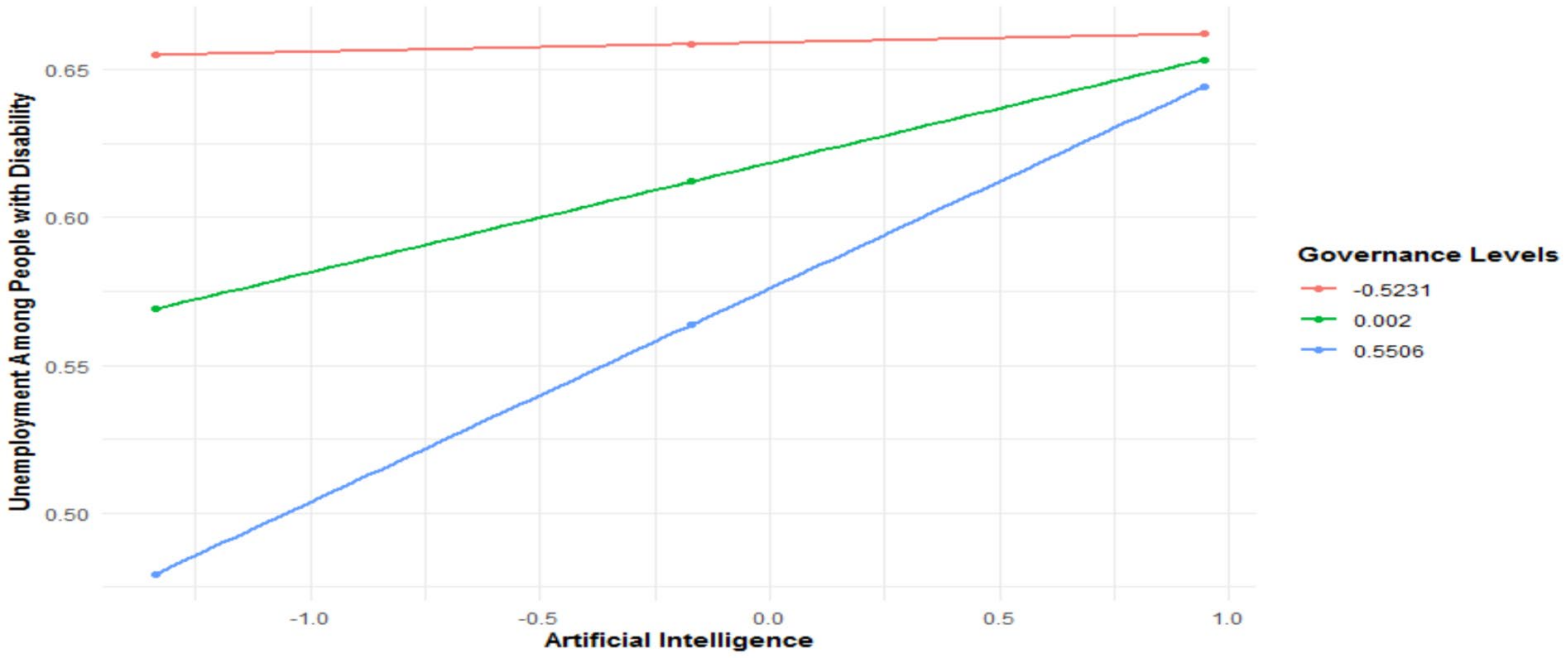
Conditional effect of AI on unemployment among people with disability in the presence of governance quality.

For individuals with an intermediate level of education, the direct effect of AI on unemployment is slightly more pronounced, with a coefficient of 0.0368 (*p* = 0.0002). This indicates that the impact of AI on unemployment is more significant in this group. Although negative, the direct effect of governance is less significant (−0.0774, *p* = 0.0584). However, the AI x Governance interaction (X*W) remains significant, with a positive coefficient of 0.0645 (*p* = 0.0142), confirming that governance intensifies the effect of AI. The interaction test (*R*^2^-chng = 0.0107, F(HC4) = 6.0683, *p* = 0.0142) strengthens this observation. As presented in Figure 7, the conditional effects show that under low governance, the effect of AI is 0.0432 (*p* = 0.0145). With medium governance, this effect rises to 0.0627 (*p* = 0.0005). Finally, under high governance, the conditional effect reaches 0.0819 (*p* = 0.0001).

**Figure 8:**
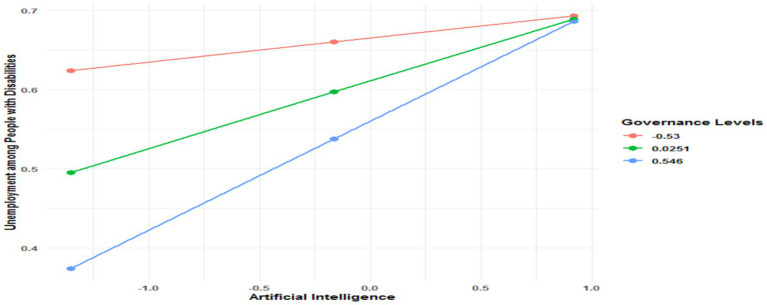
Conditional effect of AI on unemployment among people with disability in the presence of governance quality.

In the model for advanced education, the direct effect of AI is the highest among the three groups, with a coefficient of 0.083 (*p* < 0.0001), showing that individuals with an advanced level of education are the most affected by AI in terms of unemployment. Governance has a significant and negative direct effect on unemployment (−0.0977, *p* = 0.0436). However, the AI x Governance interaction (X*W), with a coefficient of 0.0994 (*p* < 0.0001), highlights an even stronger amplification of the effect of AI in this group. The interaction test (*R*^2^-chng = 0.0466, F (HC4) = 11.5424, *p* = 0.0007) confirms this amplification. As demonstrated in Figure 8, the conditional effects show that under low governance, the effect of AI is 0.1426 (*p* = 0.0035). With medium governance, this effect rises to 0.1934 (*p* < 0.0001). Finally, under high governance, the effect reaches 0.2442 (*p* < 0.0001).

In summary, these results indicate that the effect of AI on unemployment increases with the level of education, being particularly pronounced for individuals with advanced education. Although generally contributing to reducing unemployment, governance plays a paradoxical role: it amplifies the negative effects of AI in all three models analyzed, with increasing intensity depending on the level of education. These observations contradict hypothesis H3.2, as although governance does moderate the association between AI and unemployment for people with disabilities, this moderation does not reduce the negative effects of AI. On the contrary, it exacerbates them, suggesting that in the context of strong governance, policies favoring the adoption and integration of AI and reinforced infrastructures may intensify the negative impacts of AI on employment, particularly for the most qualified individuals. Thus, H4 is confirmed in terms of moderation but contradicted regarding the potential attenuation of negative effects. These results align with the work of Saba and Ngepah (31), who show that governance, while essential for supporting economic growth and employment, can also reinforce the unequal effects of AI. Leontief (32) had already warned about the potential impact of technological advancements on income distribution, a finding that remains relevant in AI. Furthermore, the work of Lee et al. (33) suggests that, in contexts of strong governance, incentives for innovation can sometimes exacerbate inequalities, particularly by reinforcing disparities in access to employment in highly technological sectors.

#### 4.2.4 Conditional indirect impact of AI on unemployment among people with disability

Tables 9–11 present the conditional indirect impacts of AI on unemployment across three levels of education (basic, intermediate, and advanced) while accounting for the role of governance. Each table displays the moderated mediation index, which measures the impact of governance on the link between AI and unemployment through education. According to Hayes’s (57) recommendations, a moderated mediation index is considered significant if the confidence interval does not contain zero. If the confidence interval includes zero, the index is not significant. Table 9 shows the indirect effects of AI on the unemployment of people with disabilities through basic education, according to governance levels. The moderated mediation index for governance is −0.0020, with a confidence interval of [−0.0104; 0.0048]. Since this interval includes zero, the index is insignificant, indicating that governance does not have a relevant moderating effect on the link between basic education, AI, and unemployment. The conditional indirect effects vary by governance level but remain non-significant. For low governance, the indirect effect is 0.0053 ([0.0000; 0.0141]), while for medium governance, it is 0.0043 ([0.0005; 0.0101]). Finally, for high governance, the indirect effect is 0.0031 ([−0.0006; 0.0096]). The inclusion of zero in these intervals indicates that these effects are not significant. Table 10 presents the indirect impact of AI on unemployment via intermediate education, depending on the quality of governance. The moderated mediation index for governance is −0.0004, with a confidence interval of [−0.0082; 0.0063]. Since this interval includes zero, the mediation index is insignificant, indicating the absence of a moderating effect of governance on the relationship between intermediate education, AI, and unemployment. The conditional indirect effects for different governance levels are also non-significant. For low governance, the indirect effect is 0.0003 ([−0.0052; 0.0065]), for medium governance, it is 0.0001 ([−0.0025; 0.0029]), and for high governance, it reaches −0.0001 ([−0.0035; 0.0027]). These intervals, including zero, render these effects non-significant. Table 11 highlights a significant change. The moderated mediation index for governance is −0.0361 ([−0.0658; −0.0168]), confirming its significance. Regarding the conditional indirect effects, low governance results in an effect of −0.0262 ([−0.0483; −0.0061]), indicating that AI contributes to a notable reduction in unemployment for people with disabilities through advanced education. With medium governance, this effect strengthens to −0.0462 ([−0.0714; −0.0274]), further accentuating the reduction in unemployment. Finally, high governance amplifies this effect even further, reaching −0.0651 ([−0.0973; −0.0397]), representing the most significant and maximal effect on reducing unemployment for people with disabilities through advanced education. This means that high governance maximizes the positive impact of AI on reducing unemployment for people with disabilities through advanced education, representing the most significant impact among the three governance levels.

**Table 9:** Conditional indirect impacts of AI on unemployment among people with disability through basic education.

	Governance	Indirect effect	BootSE	Boot LLCI	Boot ULCI
Low	−0.5231	0.0053	0.0037	0.0000	0.0141
Middle	0.002	0.0043	0.0025	0.0005	0.0101
High	0.5506	0.0031	0.0026	−0.0006	0.0096
Moderated mediation index					
	Index	Boot SE	Boot LLCI	Boot ULC	
Governance	−0.0020	0.0037	−0.0104	0.0048	

Lower = 16th percentile; Middle: 50th percentile; Upper: 84th percentile.

**Table 10:** Conditional indirect impact of AI on unemployment among people with disability through intermediate education.

	Governance	Indirect effect	Boot SE	Boot LLCI	Boot ULCI
Low	−0.5231	0.0003	0.0028	−0.0052	0.0065
Middle	0.0020	0.0001	0.0013	−0.0025	0.0029
High	0.5506	−0.0001	0.0015	−0.0035	0.0027
Moderated mediation index					
	Index	Boot SE	Boot LLCI	Boot ULCI	
Governance	−0.0004	0.0034	−0.0082	0.0063	

Lower = 16th percentile; Middle: 50th percentile; Upper: 84th percentile.

**Table 11:** Conditional indirect effects of AI on unemployment among people with disability through advanced education.

	Governance	Indirect effect	BootSE	Boot LLCI	Boot ULCI
Low	−0.5231	−0.0262	0.0105	−0.0483	−0.0061
Middle	0.0020	−0.0462	0.0111	−0.0714	−0.0274
High	0.5506	−0.0651	0.0148	−0.0973	−0.039
Moderated mediation index					
	Index	Boot SE	Boot LLCI	Boot ULCI	
Governance	−0.0361	0.0123	−0.0658	−0.0168	

Lower = 16th percentile; Middle: 50th percentile; Upper: 84th percentile.

The results presented partially confirm hypothesis H4, which suggests that the quality of governance influences the impact of artificial intelligence on unemployment through educational pathways. While the indirect effects of AI on unemployment through basic and intermediate education are not significant, the analyses show that, in the context of advanced education, higher-quality governance significantly amplifies the reduction of unemployment among people with disabilities. Indeed, the moderated mediation index and conditional indirect effects reveal that the positive impact of AI on reducing unemployment is maximized under high governance, thus highlighting the importance of a strong institutional environment to maximize the benefits of AI through education in this context.

**Figure 9:**
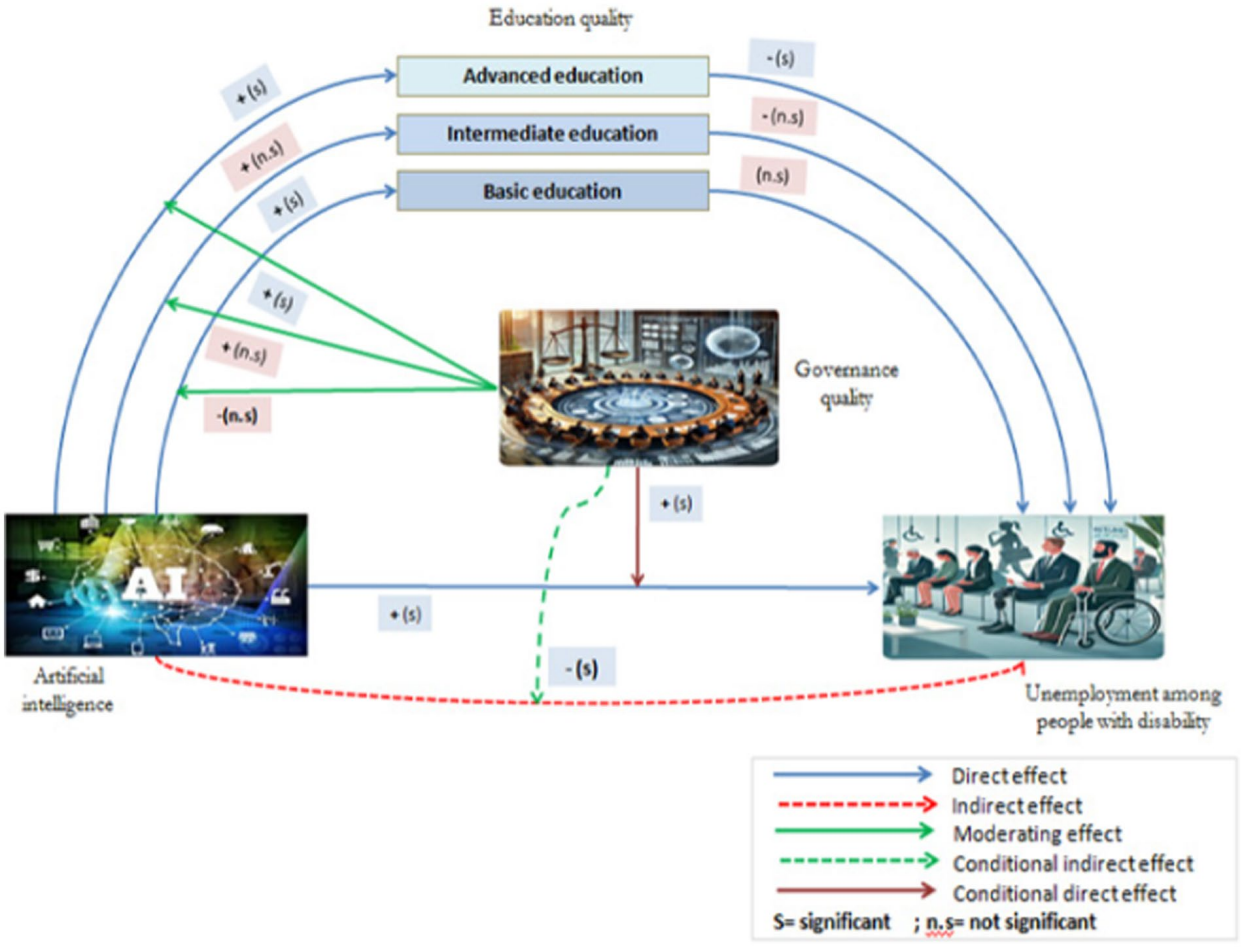
Summary of the obtained results. Conditional indirect effect is significant only for advanced education.

Figure 9 summarizes the results and shows that AI has a dual impact on unemployment among people with disabilities, moderated by governance quality and mediated through education levels. The direct effect of AI on unemployment is significant and positive, indicating that automation and insufficiently tailored technologies exacerbate job losses. The indirect effects via education reveal contrasting outcomes: AI has negligible impact on unemployment through basic and intermediate education, as these levels do not equip individuals with the advanced skills demanded in a tech-driven labor market. However, advanced education significantly reduces unemployment, with AI-enhanced tools providing specialized training and fostering employability in high-demand sectors. Governance quality plays a pivotal yet paradoxical role, amplifying the positive impact of AI on advanced education while simultaneously intensifying its negative direct effects on unemployment. This paradox arises because governance frameworks often prioritize technological advancements without adequately addressing employment disparities. The findings underscore the critical need for robust governance structures and targeted policies to harness AI’s potential in fostering inclusive labor markets. In conclusion, advanced education supported by AI and effective governance emerges as a key strategy for reducing unemployment among disabled individuals, but ensuring equity and inclusivity requires careful alignment of technological progress with inclusive policy frameworks.

## 5 Conclusion and policy implications

In the context of accelerated digital transformation and growing automation, the integration of AI into the labor market presents major challenges, particularly regarding the inclusion of vulnerable groups, such as people with disabilities. These issues raise the question of how to effectively mobilize AI to reduce socio-economic inequalities and promote fair participation in the labor market. This study analyzes how AI influences unemployment within this population, highlighting the central role of education and governance in this process in 27 high-tech developed countries. The main objective of this research is to assess the impact of AI on the unemployment of people with disabilities, considering the mediating effect of education and the moderating role of governance. We used a moderated mediation methodology (Model 8 of Hayes PROCESS Macro) to achieve this objective. This rigorous methodological approach provides an in-depth understanding of the complex interactions between AI, education, and governance while shedding light on dynamics that could inform public and institutional policies.

The study explores several key hypotheses concerning the impact of AI on the unemployment of people with disabilities. Regarding hypothesis 1, which posited that AI would directly reduce the unemployment of disabled individuals, the results show that contrary to expectations, the direct effect of AI is significant but positive, indicating an initial increase in unemployment. This is likely due to the automation of tasks and the mismatch of new technologies with the specific needs of this population. Hypothesis 2, a mediation hypothesis, suggested that AI could reduce the unemployment of people with disabilities by improving the quality of education. This hypothesis is partially confirmed. The results reveal that advanced education, supported by AI, is crucial in reducing unemployment, while basic and intermediate education does not have a significant effect. Regarding hypothesis 3.1, governance is confirmed as a key factor in amplifying the effects of AI on advanced education, although it does not have a significant moderating impact on basic and intermediate education. For hypothesis 3.2, the results show that while governance generally reduces unemployment, it paradoxically amplifies the negative effects of AI on the unemployment of people with disabilities across different levels of education, partially refuting this hypothesis. Finally, hypothesis 4 is partially confirmed: high-quality governance determines the extent to which AI reduces the unemployment of disabled individuals through advanced education, maximizing benefits in a robust institutional environment. These results reveal the need for an integrated approach, combining expanded access to advanced education, digital skills training, and a strong institutional environment. Together, these elements form a critical foundation for transforming the challenges posed by AI into inclusive and sustainable opportunities.

The findings of this study offer several important policy implications for governments to maximize the benefits of AI while reducing socio-economic inequalities, particularly for people with disabilities. First, governments should develop inclusive policies that ensure AI technologies are adapted to the specific needs of disabled individuals. This includes designing accessible software and tools, as well as establishing accessibility standards for new technologies. Moreover, it is essential to promote responsible automation that does not replace jobs accessible to disabled people but complements and enhances them. Next, strengthening education and training is crucial. The results show that advanced education plays a critical role in reducing the unemployment of people with disabilities. Therefore, governments should facilitate access to specialized training tailored to the needs of the digital labor market. It is also important to establish continuous training programs to help people with disabilities acquire new skills and adapt to technological changes. Governance emerges as a key factor in this context. High-quality governance is essential to maximize the benefits of AI. Governments must establish clear and transparent policies to regulate AI use, ensuring it is used ethically and inclusively. Collaboration between the public and private sectors should be encouraged to develop inclusive initiatives and support programs for people with disabilities. Promoting inclusive innovation is also important. Governments can offer tax incentives and subsidies to companies developing inclusive technologies and employing people with disabilities. It is also crucial to invest in research and development to create technological solutions that meet the needs of disabled individuals and promote their inclusion in the labor market. Finally, it is essential to establish monitoring and evaluation mechanisms to assess the impact of policies and programs on the inclusion of people with disabilities in the labor market.

This study also presents certain limitations that should be considered to guide future research. First, the artificial intelligence indicator is limited to industrial robot installations, which do not fully capture the diversity of AI technologies, such as machine learning systems or digital platforms, that could influence the employment of people with disabilities. A broader analysis incorporating these other forms of AI could provide a more comprehensive view. Second, the sample is restricted to 27 developed, high-tech countries, limiting the generalizability of the findings. This excludes the realities of developing countries, where infrastructure and needs differ. Additionally, the global governance index used does not distinguish the specific effects of its components, such as corruption or government effectiveness, which could differently modulate AI’s impact. The study also does not account for potential gender differences in how AI affects employment outcomes for people with disabilities, which sectoral employment patterns, social norms, or access to adaptive technologies could influence. Finally, the model focuses solely on education as a mediator, overlooking other potential channels, such as lifelong learning or technological adaptation programs. For future research, these limitations open several avenues. We could extend the analysis to developing countries to examine how AI affects the employment of people with disabilities in less technologically advanced contexts. A sector-specific study could also clarify differences between areas vulnerable to automation and those conducive to inclusion. Exploring the role of targeted policies, such as vocational training programs for people with disabilities, could better mitigate AI’s negative effects. Lastly, investigating gender disparities in AI’s impact on employment would provide deeper insights into how different demographic groups experience technological change, enriching recommendations for inclusive labor policies.

## Data Availability

Publicly available datasets were analyzed in this study. This data can be found here: The datasets supporting the findings of this study are publicly available from the following sources: ILOSTAT (https://ilostat.ilo.org), Our World in Data (https://ourworldindata.org), IFR (https://ifr.org), and WGI (https://worldbank.org).

## Footnotes

^1^Austria, Belgium, the Czech Republic, Denmark, Estonia, Finland, France, Germany, Greece, Hungary, Iceland, Ireland, Italy, Latvia, Lithuania, Malta, the Netherlands, Norway, Poland, Portugal, Slovakia, Slovenia, Spain, Sweden, Switzerland, the United Kingdom, and the United States.
